# Expression of NF-κB Isoforms and IKK Complex Subunits Differs in Peripheral Blood Mononuclear Cells (PBMCs) of Patients with Meningiomas: A Pilot Study

**DOI:** 10.3390/life16060880

**Published:** 2026-05-24

**Authors:** Ewa Kowalewska, Joanna Kamińska, Marta Żebrowska-Nawrocka, Ewa Balcerczak, Magdalena Rybaczek, Tomasz Łysoń, Marzena Tylicka, Natalia Wawrusiewicz-Kurylonek, Joanna Matowicka-Karna, Olga Martyna Koper-Lenkiewicz

**Affiliations:** 1 Department of Clinical Laboratory Diagnostics, Clinical Hospital of the Medical University of Bialystok, 15A Jerzego Waszyngtona St., 15-269 Bialystok, Poland; joanna.kaminska@umb.edu.pl (J.K.); joanna.matowicka-karna@umb.edu.pl (J.M.-K.); 2 Department of Clinical Laboratory Diagnostics, Medical University of Bialystok, 15A Jerzego Waszyngtona St., 15-269 Bialystok, Poland; 3 Department of Pharmaceutical Biochemistry and Molecular Diagnostics, Medical University of Lodz, 1 Muszynskiego St., 90-151 Lodz, Poland; marta.zebrowska@umed.lodz.pl (M.Ż.-N.); ewa.balcerczak@umed.lodz.pl (E.B.); 4 Laboratory of Molecular Diagnostics, Brain Laboratories, Medical University of Lodz, 4 Czechoslowacka St., 92-216 Lodz, Poland; 5 Department of Neurosurgery, Clinical Hospital of the Medical University of Bialystok, 24A M. Sklodowskiej-Curie St., 15-276 Bialystok, Poland; magdalenarybaczek@interia.pl (M.R.); tomasz.lyson@umb.edu.pl (T.Ł.); 6 Department of Medical Biophysics, Medical University of Bialystok, Adama Mickiewicza 2a St., 15-222 Bialystok, Poland; marzena.tylicka@umb.edu.pl; 7 Department of Clinical Genetics, Medical University of Białystok, Waszyngtona 13 St., 15-089 Bialystok, Poland; natalia.wawrusiewicz-kurylonek@umb.edu.pl

**Keywords:** IKK complex, meningioma, NF-κB pathway, peripheral blood mononuclear cells (PBMCs), real-time RT-PCR

## Abstract

Introduction: The NF-κB signaling pathway is a key regulator of oncogenic processes; however, its systemic role in meningiomas remains poorly understood. The aim of this pilot study was to evaluate the expression of genes encoding NF-κB isoforms and IKK complex subunits in peripheral blood mononuclear cells (PBMCs) of patients with meningiomas prior to tumor resection. Methods: The study included 31 patients with meningiomas (WHO grades G1-G3) and 18 healthy volunteers. PBMCs were isolated using density gradient centrifugation, and total RNA was extracted. mRNA expression levels of NFKB1, NFKB2, RELA, RELB, c-REL, CHUK, IKBKB, and IKBKG were quantified by real-time PCR, with GAPDH used as the reference gene. Results: In patients with meningiomas, significantly lower expression of NFKB1 and higher expression of RELA, CHUK, and IKBKB were observed compared with the control group. NFKB1 expression was significantly higher in patients with higher tumor grades (WHO G2/G3) than in those with grade G1 tumors. Moreover, male patients exhibited higher expression levels of c-REL, CHUK, and IKBKB than female patients. Strong positive correlations were observed between components of the canonical NF-κB pathway. Discussion: The results may indicate systemic dysregulation of the NF-κB pathway in immune cells of patients with meningiomas, potentially characterized by activation of the canonical pathway and a shift toward p65/p65 homodimer formation. These alterations could reflect mechanisms associated with immunosuppression. NFKB1 expression may warrant further investigation as a candidate peripheral biomarker of tumor aggressiveness, while the observed sexual dimorphism in gene expression might suggest that sex could represent a relevant factor, requiring confirmation in prospective studies.

## 1. Introduction

Meningiomas constitute the most common primary neoplasm of the central nervous system, accounting for approximately 38% of all tumors in this location worldwide [1]. According to the World Health Organization (WHO) classification, three grades of malignancy are distinguished: benign meningiomas (WHO grade I), atypical meningiomas (WHO grade II), and anaplastic/malignant meningiomas (WHO grade III) [2].

**Figure 1:**
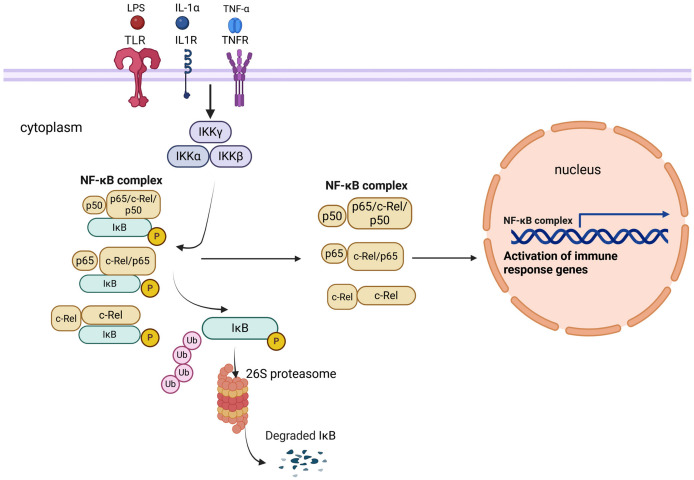
Schematic representation of the canonical (classical) NF-κB activation pathway. Activation of the canonical NF-κB pathway is initiated by the binding of ligands such as TNF-α, IL-1α, or LPS to their respective membrane receptors. Receptor stimulation leads to activation of the IKK complex, composed of the IKKα (CHUK), IKKβ, and IKKγ (NEMO) subunits. The IKK complex phosphorylates inhibitory IκB proteins associated with NF-κB dimers (most commonly p65/p50, p65/c-Rel, or c-Rel/p50), thereby triggering their polyubiquitination and subsequent degradation by the 26S proteasome. Degradation of IκB enables the translocation of released NF-κB dimers into the nucleus, where they bind to DNA and activate transcription of genes regulating the immune response. Figure was created with BioRender.

**Figure 2:**
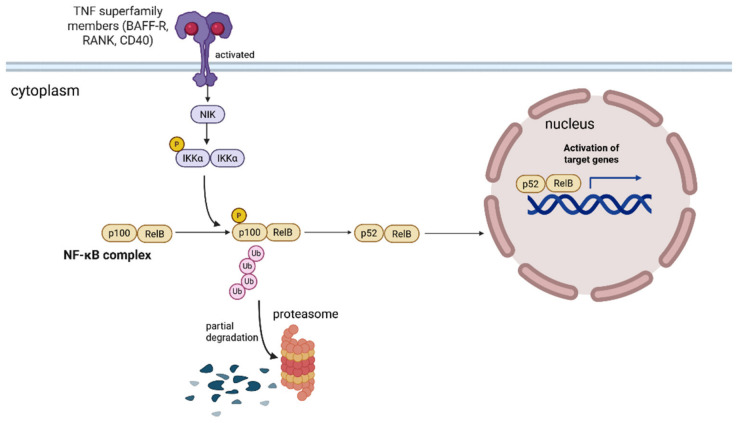
Schematic representation of the non-canonical (alternative) NF-κB activation pathway. Activation of the non-canonical NF-κB pathway is initiated by stimulation of selected receptors from the TNF receptor family, such as BAFF-R, CD40, or RANK, leading to activation of NF-κB-inducing kinase (NIK) in the cytoplasm. NIK phosphorylates and activates IKKα, which in turn catalyzes the phosphorylation of the p100 precursor within the NF-κB complex composed of p100 and RelB. Under resting conditions, p100 functions as an inhibitor by retaining RelB in the cytoplasm. Upon phosphorylation, p100 undergoes ubiquitination and partial degradation by the 26S proteasome, which removes its C-terminal region, resulting in the formation of the active p52 subunit. The p52-RelB complex subsequently translocates to the nucleus, where it binds to DNA and initiates transcription of target genes. Figure was created with BioRender.

One of the key regulators of cellular processes involved in tumorigenesis is the transcription factor NF-κB (nuclear factor kappa-light-chain-enhancer of activated B cells), which controls the expression of genes responsible for proliferation, cell survival, inflammatory responses, and the development of treatment resistance. The NF-κB protein family comprises five subunits: NF-κB1 (p50, precursor p105), NF-κB2 (p52, precursor p100), RelA (p65), RelB, and c-Rel, which form homo- and heterodimers with diverse transcriptional activities. Activation of the NF-κB pathway occurs primarily through the canonical (classical) and non-canonical (alternative) mechanisms, which differ in both the molecular composition of protein complexes and the type of initiating stimulus [3,4,5] (Figure 1 and Figure 2).

Aberrant activation of the NF-κB pathway has been documented in numerous malignancies, including gastric cancer [6], colorectal cancer [7], breast cancer [8], lung cancer [9], lymphomas [10], and gliomas [11]. In the context of these diseases, NF-κB promotes neoplastic transformation by stimulating proliferation, inhibiting apoptosis, inducing neoangiogenesis, and facilitating invasion and metastasis [3]. Moreover, NF-κB activation contributes to the remodeling of the tumor microenvironment by sustaining chronic inflammation and immunosuppressive mechanisms, thereby promoting disease progression and limiting therapeutic efficacy [12,13,14,15].

In contrast to other malignancies, the role of NF-κB in the biology of meningiomas remains insufficiently characterized. Available data indicate a potential association between NF-κB2 subunit expression and unfavorable prognosis. High expression of PD-L1 (programmed death-ligand 1) in meningioma tissue has been shown to correlate with shorter recurrence-free survival and to co-occur with increased NF-κB2 expression, independently of WHO grade and extent of tumor resection [16]. An association has also been demonstrated between PD-L1 expression and NF-κB2 levels, as well as its induction under hypoxic conditions, suggesting a potential role for NF-κB2 in regulating immunological and adaptive mechanisms of the tumor, including the cellular response to hypoxia [16].

A study by Maalim et al. [17], conducted on the IOMM-LEE and CH157-MN cell lines representing high-grade meningiomas, demonstrated that activation of the canonical NF-κB pathway is closely associated with the RACK1–CSNK2B axis. The RACK1 protein binds the β subunit of casein kinase 2 (CSNK2B), protecting it from ubiquitin-dependent degradation, thereby stabilizing the CK2 complex and leading to enhanced NF-κB transcriptional activity. Silencing of RACK1 significantly reduced NF-κB activity, whereas CSNK2B overexpression augmented it. Stabilization of the CK2 complex correlated with increased levels of phosphorylated p65 (p-p65) and elevated expression of subunits of the IKK enzymatic complex, which activates the NF-κB pathway. Furthermore, NF-κB activation induced the transcription of cell cycle-regulatory genes such as CDK4 and CCND3 (cyclin D3), accelerating G2/M phase progression and enhancing proliferation and migration of meningioma cells [17].

Despite these observations, the detailed molecular mechanisms underlying NF-κB activation in meningiomas and their systemic significance, including in the context of the immune response, remain incompletely understood. In particular, there is a lack of data regarding the activity of NF-κB pathway components in immune cells of patients with meningiomas. Therefore, the aim of the present pilot study was to evaluate the expression of *NFKB* isoform genes and subunits of the *IKK* complex in peripheral blood mononuclear cells (PBMCs) of patients with meningioma prior to surgical tumor resection, compared with individuals without neoplastic disease. To the best of our knowledge, this is the first study to evaluate mRNA expression of *NF-κB* isoforms and *IKK* complex subunits in PBMCs of patients with meningiomas. Although descriptive in nature, our approach aligns with the emerging concept of peripheral immunoprofiling in neuro-oncology, where PBMCs serve as a minimally invasive surrogate for tumor–-host immune interactions [18,19]. In this context, exploratory and pilot studies play an important role, particularly in relatively rare diseases, by generating testable hypotheses for subsequent mechanistic and clinical investigations. The results obtained may contribute to a better understanding of the role of the NF-κB pathway in systemic mechanisms accompanying meningioma development and may identify potential biomarkers of prognostic or therapeutic relevance.

## 2. Materials and Methods

### 2.1. Patients

All procedures were carried out in full compliance with the ethical standards of the Declaration of Helsinki. The study protocol received ethical clearance from the Bioethics Human Research Committee at the Medical University of Bialystok (Approval No. APK.002.357.2022), and written informed consent was obtained from each participant before study entry.

The study group included 31 patients diagnosed with meningioma (21 women and 10 men) who underwent surgical tumor resection at the Department of Neurosurgery, Clinical Hospital of the Medical University of Bialystok. The median age of the patients was 64 years (IQR: 52–71 years, range: 27–88 years). Demographic, clinical, and laboratory characteristics of the meningioma group are summarized in Table 1.

Female meningioma patients had a significantly lower median creatinine concentration (0.66 mg/dL; interquartile range: 0.61–0.74 mg/dL) compared to male meningioma patients (0.82 mg/dL; interquartile range: 0.77–0.84 mg/dL) (*p* = 0.0077) (Table S1). Meningioma patients with higher WHO grades (G2 and G3) had a significantly higher median glucose concentration (151 mg/dL; interquartile range: 128–156 mg/dL) compared to patients with a lower WHO grade (G1) (99 mg/dL; interquartile range: 86–1233 mg/dL) (*p* = 0.0413). Meningioma patients with higher WHO grades (G2 and G3) had a significantly lower median creatinine concentration (0.52 mg/dL; interquartile range: 0.39–0.66 mg/dL) compared to patients with a lower WHO grade (G1) (0.73 mg/dL; interquartile range: 0.66–0.83 mg/dL) (*p* = 0.0188) (Table S2). Meningioma patients with a median age higher than 64 years had a significantly higher median glucose concentration (123 mg/dL; interquartile range: 98–150 mg/dL) compared to those of ≤64 years (90 mg/dL; interquartile range: 84–104 mg/dL) (*p* = 0.0171) (Table S3).

The control group consisted of 18 healthy volunteers, including 9 women and 9 men, with a median age of 50 years (IQR: 36–57 years, range: 24–85 years).

Exclusion criteria were strictly applied to both study groups and included the presence of neurodegenerative diseases (e.g., multiple sclerosis), a history of neuro-infections or brain tumors, recent surgical procedures or major trauma within the preceding months, as well as the use of antibiotics, anti-inflammatory medications, or corticosteroids within the last 30 days.

### 2.2. Sample Collection and Storage

Whole venous blood samples were collected into 9 mL K_3_EDTA tubes (1.6 mg EDTA/mL; catalog no. 02.1066.001, S-Monovette, SARSTEDT, Nümbrecht, Germany) for the isolation of peripheral blood mononuclear cells (PBMCs). Isolation of PBMCs was initiated within 30 min of blood collection using Histopaque (catalog no. 10771, Sigma Life Science, Darmstadt, Germany). All samples were stored at −80 °C until further analysis, and experimental procedures were carried out immediately after thawing.

### 2.3. Peripheral Blood Mononuclear Cell (PBMC) Isolation Using Density Gradient Centrifugation with Ficoll Histopaque

PBMCs were isolated using a density gradient centrifugation method with Ficoll-Paque (Histopaque, catalog no. 10771, Sigma Life Science), following the steps below:

- Three milliliters of Ficoll Histopaque were added to each of three sterile 10 mL centrifuge tubes.
- Subsequently, 3 mL of freshly collected blood was carefully layered onto the Ficoll using a 1 mL pipette, ensuring minimal disturbance to maintain clear separation between the layers.
- The tubes were centrifuged immediately at 400× *g* for 30 min at 20 °C without applying the brake.
- The mononuclear cell layer (whitish buffy coat, approx. 1.5 mL) was aspirated from the plasma–Ficoll interface using a sterile pipette.
- Cells were washed three times in sterile phosphate-buffered saline (PBS) by centrifugation at 250× *g* for 10 min each.
- The resulting PBMC pellet was resuspended in 600 µL of RTL buffer containing β-mercaptoethanol, thoroughly mixed, aliquoted into two sterile microcentrifuge tubes, and stored at −80 °C until RNA extraction [20].

### 2.4. RNA Isolation and Reverse Transcription

Total RNA was isolated from PBMC samples—comprising T cells, B cells, NK cells, monocytes, and dendritic cells—using the RNeasy Mini Kit (catalog no. 74104, Qiagen GmbH, Hilden, Germany) following the manufacturer’s protocol. The concentration and purity of the extracted RNA were assessed spectrophotometrically using the NanoDrop One system (Thermo Fisher Scientific, Madison, WI, USA). RNA concentrations in the meningioma group ranged from 37.6 to 344.6 ng/μL, with A260/A280 absorbance ratios between 1.7 and 2.0 considered acceptable. All RNA samples were stored at −80 °C until further analysis [20].

Complementary DNA (cDNA) was synthesized from total RNA using the High-Capacity cDNA Reverse Transcription Kit with RNase Inhibitor (catalog no. 4374966, Applied Biosystems by Thermo Fisher Scientific, Vilnius, Lithuania), following the manufacturer’s instructions. The final RNA concentration in each reaction was adjusted to 0.5 μg/μL. Reverse transcription was performed under the following thermal conditions: 25 °C for 10 min, 37 °C for 120 min, and 85 °C for 5 min. The resulting cDNA samples were stored at −20 °C until further analysis.

### 2.5. Real-Time PCR

The GAPDH gene, encoding glyceraldehyde-3-phosphate dehydrogenase, was used as the reference (housekeeping) gene. For this purpose, a commercially available primer set (QuantiTect^®^ Primer Assay, catalog no. QT01192646, Hs_GAPDH_2-SG; Qiagen GmbH, Hilden, Germany) was employed. The expression levels of the target genes—*NFKB1*, *NFKB2*, *RELA*, *RELB*, *c-REL* (catalog nos. QT00063791, QT01662997, QT02324308, QT00038640, QT00052472, respectively; QuantiTect^®^ Primer Assay, Qiagen GmbH, Hilden, Germany)—as well as *CHUK*, *IKBKB*, and *IKBKG* (primers were synthesized by Genomed S.A., Poland; the primers’ sequences were as follows: *CHUK*expF: 5′->3′: CTCCTCAAGATGGGGAGACTT, *CHUK*expR: 5′->3′: CCATAGGTTTGGGGACAGTGA; *IKBKB*expF: 5′->3′: GGAAGTACCTGAACCAGTTTGAG, *IKBKB*expR: 5′->3′: GCAGGACGATGTTTTCTGGCT; *IKBKG*expF: 5′->3′: GGGAAAGATGCTGTTCCAGGC, *IKBKG*expR: 5′->3′: CACCATCTCACACAGTTGGC) were quantified using the QuantStudio™ 5 Real-Time PCR System (Applied Biosystems by Thermo Fisher Scientific, Singapore) with PowerUp™ SYBR™ Green Master Mix (catalog no. A25741, Applied Biosystems by Thermo Fisher Scientific).

Each real-time PCR reaction was prepared in a final volume of 10 μL, containing 5 μL of PowerUp™ SYBR™ Green Master Mix (Applied Biosystems, Waltham, MA, USA), 0.5 μL of each gene-specific primer, 4 μL of nuclease-free water, and 1 μL of cDNA template. Reactions were performed in triplicate for each sample, with separate tubes used for target and reference gene amplifications. To ensure assay specificity and exclude contamination, no-template control (NTC) reactions were included in each run. The qPCR thermal cycling conditions were as follows: UNG pretreatment at 50 °C for 2 min, and initial denaturation at 95 °C for 2 min, followed by 40 amplification cycles of denaturation at 95 °C for 15 s, annealing at 60 °C for 30 s, and extension at 72 °C for 30 s. A post-amplification melting curve analysis was conducted to confirm the specificity of the PCR products. Cycle threshold (Ct) values obtained from technical triplicates were averaged. Relative gene expression levels were calculated using the ΔCt method, where ΔCt represents the difference between the mean Ct value of the target gene and the Ct value of the reference gene (GAPDH) [20,21,22].

### 2.6. Statistical Analysis

All statistical computations were carried out using STATISTICA 13.0 PL (StatSoft Inc., Tulsa, OK, USA) and GraphPad Prism 8.0 (GraphPad Software, San Diego, CA, USA). Between-group comparisons were performed with the non-parametric Mann–Whitney U test, and monotonic associations between variables were quantified using Spearman’s rank correlation coefficient. Data for continuous variables are expressed as median values accompanied by interquartile ranges (25th–75th percentile). Statistical significance was defined as a two-tailed *p*-value below 0.05.

## 3. Results

### 3.1. Expression of NFKB Isoforms and IKK Complex Subunits in PBMCs of Meningioma Patients Significantly Differs from That in Non-Tumoral Individuals

**Figure 3:**
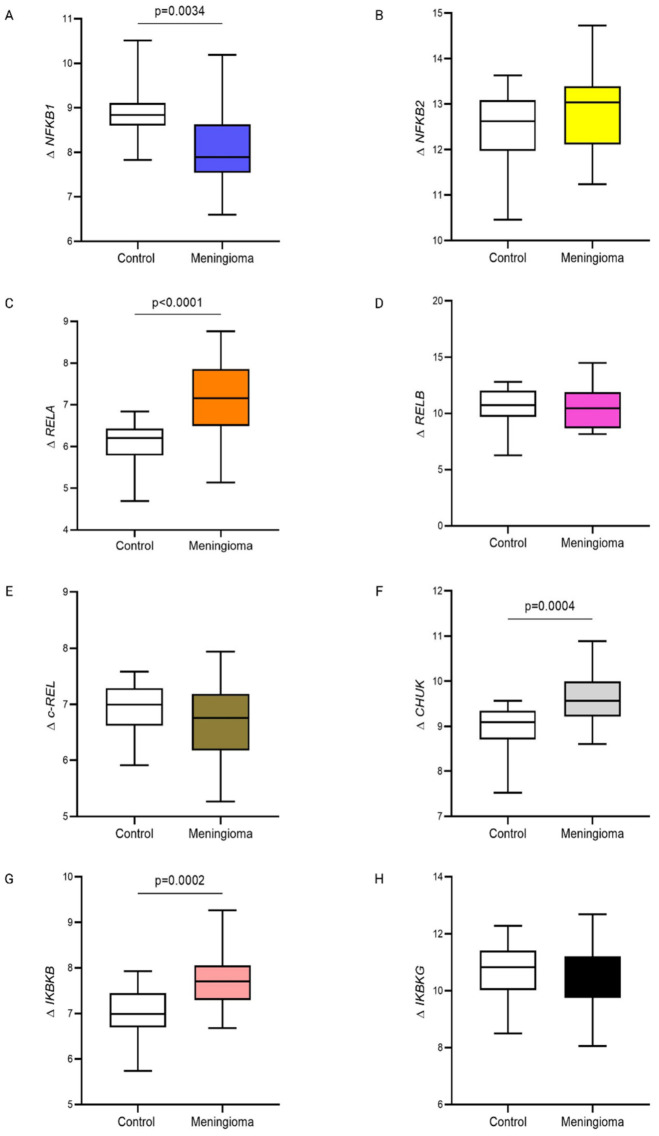
Comparison of *NF-κB* isoforms and *IKK* subunits expression in PBMCs from meningioma patients and non-tumoral individuals. (**A**) Δ*NFKB1*; (**B**) Δ*NFKB2*; (**C**) Δ*RELA*; (**D**) Δ*RELB*; (**E**) Δ*c-REL*; (**F**) Δ*CHUK*; (**G**) Δ*IKBKB*; (**H**) Δ*IKBKG*. Cycle threshold (Ct) values obtained from technical triplicates were averaged. Relative gene expression levels were calculated using the ΔCt method, where ΔCt represents the difference between the mean Ct of the target gene and the mean Ct of the reference gene (*GAPDH*). Box-and-whisker plots display interquartile ranges with median values, and whiskers indicate the full data range (minimum to maximum). A *p*-value of <0.05 (two-tailed) was considered statistically significant. IKK—I kappa B kinase; PBMCs—peripheral blood mononuclear cells; NFKB1—nuclear factor kappa B subunit 1; NFKB2—nuclear factor kappa B subunit 2; RELA—RELA Proto-Oncogene; RELB—RELB Proto-Oncogene; c-REL—REL Proto-Oncogene; CHUK—conserved helix–loop–helix ubiquitous kinase; IKBKB—inhibitor of nuclear factor kappa B kinase subunit beta; IKBKG—inhibitor of nuclear factor kappa B kinase subunit gamma.

The expression of *NFKB1* in PBMCs from meningioma patients was significantly lower than that in PBMCs from healthy, non-tumoral individuals. In contrast, the expression of *RELA* was significantly higher in PBMCs from meningioma patients compared to healthy controls. Among the *IKK* complex subunits, the expression levels of *CHUK* and *IKBKB* were also significantly higher in PBMCs from meningioma patients than in those from healthy, non-tumoral individuals (Figure 3).

### 3.2. Expression of NFKB Isoforms and IKK Complex Subunits in PBMCs of Meningioma Patients Differ Depending on Patients’ Sex and Histopathological Examination Results

**Figure 4:**
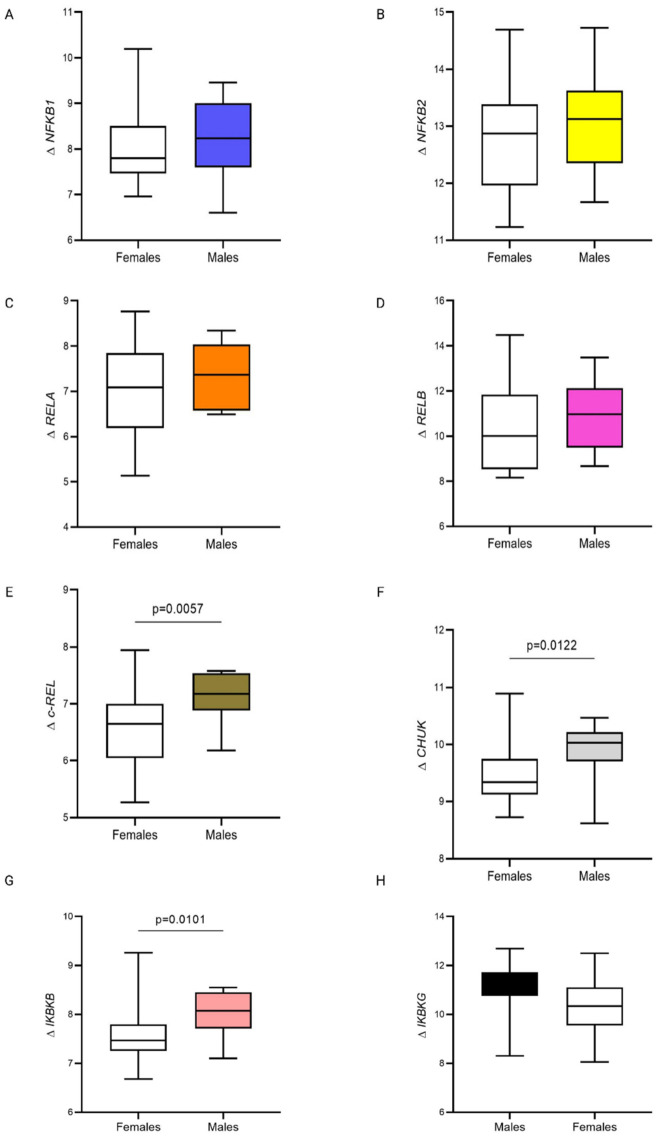
Sex-dependent expression of *NF-κB* isoforms and *IKK* subunits in PBMCs from meningioma patients. (**A**) Δ*NFKB1*; (**B**) Δ*NFKB2*; (**C**) Δ*RELA*; (**D**) Δ*RELB*; (**E**) Δ*c-REL*; (**F**) Δ*CHUK*; (**G**) Δ*IKBKB*; (**H**) Δ*IKBKG*. Cycle threshold (Ct) values obtained from technical triplicates were averaged. Relative gene expression levels were calculated using the ΔCt method, where ΔCt represents the difference between the mean Ct of the target gene and the mean Ct of the reference gene (*GAPDH*). Box-and-whisker plots display interquartile ranges with median values, and whiskers indicate the full data range (minimum to maximum). A *p*-value of <0.05 (two-tailed) was considered statistically significant. IKK—I kappa B kinase; PBMCs—peripheral blood mononuclear cells; NFKB1—nuclear factor kappa B subunit 1; NFKB2—nuclear factor kappa B subunit 2; RELA—RELA Proto-Oncogene; RELB—RELB Proto-Oncogene; c-REL—REL Proto-Oncogene; CHUK—conserved helix–loop–helix ubiquitous kinase; IKBKB—inhibitor of nuclear factor kappa B kinase subunit beta; IKBKG—inhibitor of nuclear factor kappa B kinase subunit gamma; *p*—*p*-value.

**Figure 5:**
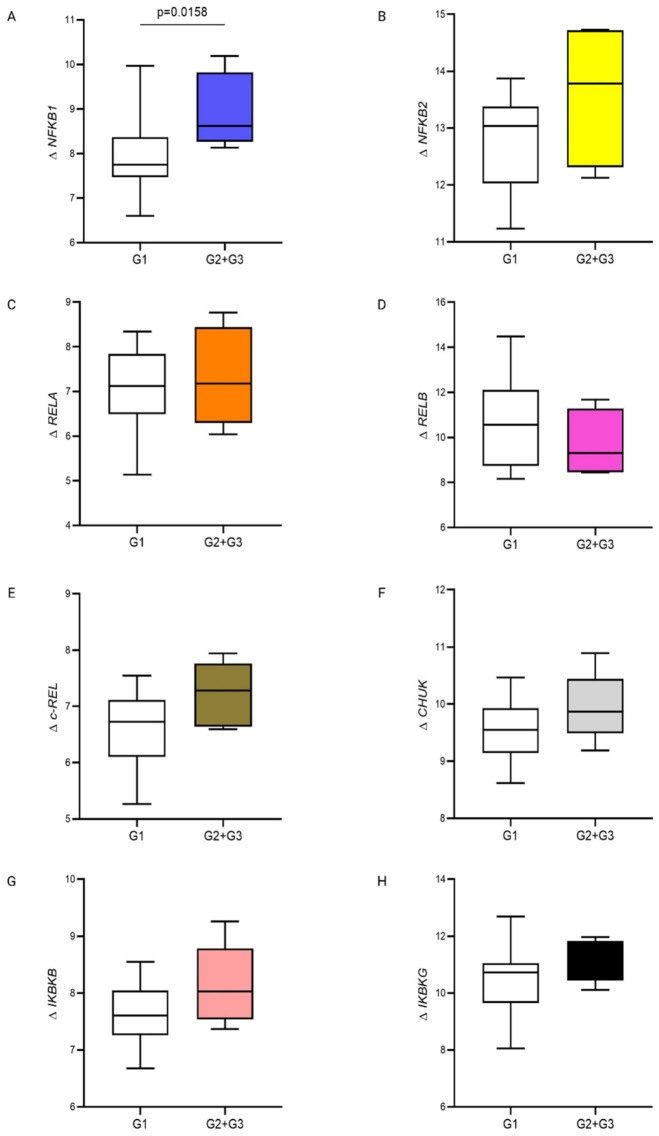
Comparison of *NF-κB* isoform and *IKK* subunit expression in PBMCs of meningioma patients according to World Health Organization (WHO) grade. (**A**) Δ*NFKB1*; (**B**) Δ*NFKB2*; (**C**) Δ*RELA*; (**D**) Δ*RELB*; (**E**) Δ*c-REL*; (**F**) Δ*CHUK*; (**G**) Δ*IKBKB*; (**H**) Δ*IKBKG*. Cycle threshold (Ct) values obtained from technical triplicates were averaged. Relative gene expression levels were calculated using the ΔCt method, where ΔCt represents the difference between the mean Ct of the target gene and the mean Ct of the reference gene (*GAPDH*). Box-and-whisker plots display interquartile ranges with median values, and whiskers indicate the full data range (minimum to maximum). A *p*-value of <0.05 (two-tailed) was considered statistically significant. IKK—I kappa B kinase; PBMCs—peripheral blood mononuclear cells; NFKB1—nuclear factor kappa B subunit 1; NFKB2—nuclear factor kappa B subunit 2; RELA—RELA Proto-Oncogene; RELB—RELB Proto-Oncogene; c-REL—REL Proto-Oncogene; CHUK—conserved helix–loop–helix ubiquitous kinase; IKBKB—inhibitor of nuclear factor kappa B kinase subunit beta; IKBKG—inhibitor of nuclear factor kappa B kinase subunit gamma; *p*—*p*-value.

The PBMCs of male meningioma patients had significantly higher levels of expression of *c-REL* compared to PBMCs of female meningioma patients. The expression of two catalytic subunits of IKK complex (*CHUK* and *IKBKB*) by PBMCs of male meningioma patients was also significantly higher compared to PBMCs of female meningioma patients (Figure 4). *NFKB1* expression in PBMCs of meningioma patients with higher WHO grades (G2 and G3) was significantly higher compared to patients with a lower WHO grade (G1) (Figure 5). Expression of *NFKB* isoforms and *IKK* complex subunits in PBMCs of meningioma patients did not differ depending on histopathological classification (Figure S1) or the age of meningioma patients (Figure S2).

### 3.3. Correlation Coefficient Analysis of NFKB Isoforms and IKK Complex Subunits

**Figure 6:**
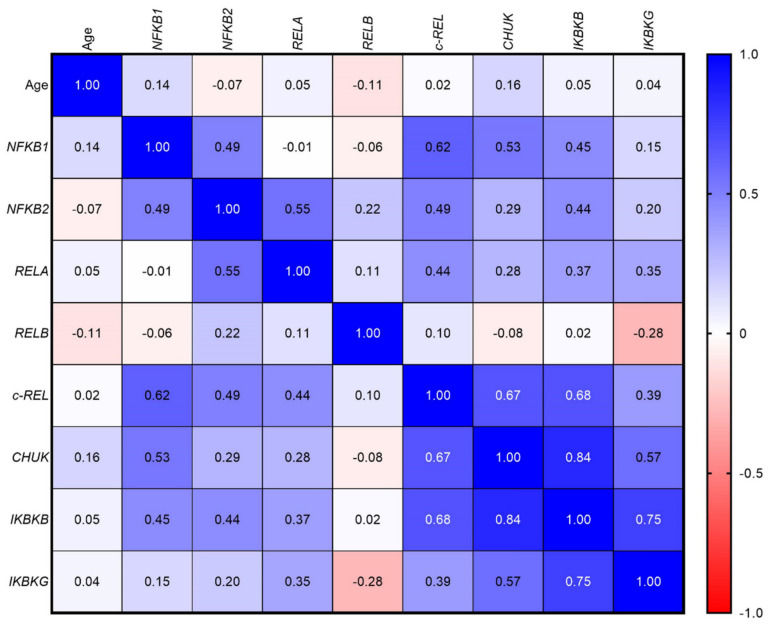
Heat map showing Spearman’s correlation coefficients among *NF-κB* isoforms, *IKK* complex subunits, and patient age. Color gradients indicate the strength and direction of correlations. IKK—I kappa B kinase; PBMCs—peripheral blood mononuclear cells; NFKB1—nuclear factor kappa B subunit 1; NFKB2—nuclear factor kappa B subunit 2; RELA—RELA Proto-Oncogene; RELB—RELB Proto-Oncogene; c-REL—REL Proto-Oncogene; CHUK—conserved helix–loop–helix ubiquitous kinase; IKBKB—inhibitor of nuclear factor kappa B kinase subunit beta; IKBKG—inhibitor of nuclear factor kappa B kinase subunit gamma.

In the first step, we correlated the expression of *NFKB1*, *NFKB2*, *RELA*, *RELB*, *c-REL*, *CHUK*, *IKBKB*, and *IKBKG* in PBMCs with patients’ age and each other. We found that *NFKB1* expression positively correlated with *NFKB2* (R = 0.49, *p* = 0.0070), *c-REL* (R = 0.62, *p* = 0.0002), *CHUK* (R = 0.53, *p* = 0.0027), and *IKBKB* (R = 0.45, *p* = 0.0109). *NFKB2* expression positively correlated with *RELA* (R = 0.55, *p* = 0.0019), *IKBKB* (R = 0.44, *p* = 0.0159) and negatively with c-REL (R= −0.49, *p* = 0.0065). *RELA* expression positively correlated with *c-REL* (R = 0.44, *p* = 0.0132) and *IKBKB* (R = 0.37, *p* = 0.0388). *c-REL* expression positively correlated with *CHUK* (R = 0.67, *p* = 0.0001), *IKBKB* (R = 0.68, *p* < 0.0001) and *IKBKG* (R = 0.39, *p* = 0.0287). *CHUK* expression positively correlated with *IKBKB* (R = 0.84, *p* < 0.0001) and *IKBKG* (R = 0.57, *p* = 0.0011). *IKBKB* expression positively correlated with *IKBKG* (R = 0.75, *p* < 0.0001). We did not find any other correlations (Figure 6, Table S5).

### 3.4. Sample Size and Post Hoc Power Analysis for Comparison Between the Meningioma Patient Group and the Control Group

The investigation of the expression of *NFKB1*, *NFKB2*, *RELA*, *RELB*, *c-REL*, *CHUK*, *IKBKB*, and *IKBKG* in PBMCs is the first in patients with tumors of the meninges. Therefore, we did not conduct a priori power analysis and sample size calculations. Post hoc power analysis and sample size calculations were conducted for all examined *NF-κB* isoforms and *IKK* complex subunits in the context of differentiating meningioma patients from non-tumoral individuals. The results varied considerably across the analyzed genes. Adequate statistical power was achieved for *NFKB1* (minimum required *n* = 12 per group; achieved power: 96%), *RELA* (*n* = 4; 100%), *CHUK* (*n* = 11; 99%), and *IKBKB* (*n* = 8; 100%), indicating that the current sample size was sufficient for detecting significant differences in expression of these targets. In contrast, substantially larger cohorts would be required to achieve adequate power for *NFKB2* (*n* = 487; achieved power: 7.5%), *c-REL* (*n* = 75; 27.5%), *IKBKG* (*n* = 1539; 5.2%), and *RELB* (*n* = 12,016; 3.2%), reflecting the limited effect sizes observed for these genes. These findings underscore the pilot nature of the study and support the need for validation in larger, prospectively recruited cohorts (Table S6).

## 4. Discussion

The role of the NF-κB pathway in meningioma pathogenesis remains poorly understood [16,17], despite its established importance in proliferation, apoptosis resistance, and immunosuppression in other malignancies, including central nervous system (CNS) tumors [11]. To our knowledge, this exploratory pilot study is the first to assess mRNA expression of *NF-κB* isoforms and *IKK* complex proteins in PBMCs from patients with meningioma.

In our pilot study, we found that expression of canonical *NF-κB* pathway isoforms and *IKK* complex kinases differs significantly between PBMCs from meningioma patients and healthy controls. Specifically, *NFKB1* (p50) expression was significantly lower in PBMCs from meningioma patients compared to healthy individuals, whereas *RELA* (p65) expression was significantly elevated. Among *IKK* complex subunits, *CHUK* (IKKα) and *IKBKB* (IKKβ) expression levels were also significantly higher in meningioma patients than in healthy controls. Moreover, we demonstrated significant positive correlations between canonical *NF-κB* pathway isoforms and *IKK* complex subunits in meningioma patients. In contrast, no correlations were observed between non-canonical pathway components.

The significantly decreased *NFKB1* (p50) expression, coupled with increased *RELA* (p65) expression, is compatible with a relative shift in dimerization equilibrium towards RelA-containing NF-κB dimers in PBMCs from meningioma patients. However, as our analyses are restricted to transcript levels, we cannot directly demonstrate changes in dimer composition, nuclear translocation or DNA-binding activity of specific NF-κB complexes, and such mechanistic interpretations should therefore be regarded as hypothesis-generating. The concurrent upregulation of *CHUK* (*IKKα*) and *IKBKB* (*IKKβ*) may indicate enhanced *IKK* complex activity, which is essential for IκB protein phosphorylation and subsequent NF-κB nuclear translocation. The observed pattern of strong correlations between key components of the canonical NF-κB pathway (C*HUK-IKBKB*, R = 0.84; *c-REL-IKBKB*, R = 0.68) may suggest coordinated activation of the signaling network.

Unlike the NFKB1 (p50) and NFKB2 (p52) subunits, RELA (p65) possesses a transactivation domain (TAD), enabling p65/p65 homodimers to directly initiate target gene transcription without requiring heterodimerization [23,24]. One of the best-characterized roles of p65/p65 homodimers involves induction of inducible nitric oxide synthase (iNOS) expression. Studies have demonstrated that in myeloid cells within the tumor microenvironment, p65/p65 and p50/p50 homodimers, rather than p50/p65 heterodimers, directly bind the *NOS2* gene promoter, activating its transcription in response to inflammatory signals such as lipopolysaccharide (LPS) and interferon-gamma (IFNγ), with p65/p65 homodimers cooperating with the IRF8 transcription factor [25]. The p65 subunit is also critical for inhibiting apoptosis and promoting tumor cell survival. In diffuse large B-cell lymphoma (DLBCL), p65 silencing proved more effective in suppressing tumor growth than targeting other NF-κB subunits, underscoring the dominant role of p65 in oncogenic processes [26].

Within the tumor microenvironment, RELA (p65) promotes immunosuppressive phenotypes and disease progression [27,28]. In tumor-associated macrophages (TAMs), p65 activity is essential for M2 polarization, directly supporting tumor growth in malignancies including glioblastoma and lung cancer [27]. RELA (p65) functions as a molecular brake on immune responses by inducing expression of the inhibitory molecule B7-H4 (B7x), which suppresses cytotoxic CD8+ T lymphocyte (CTL) activity through direct cell-to-cell contact. Simultaneously, p65 indirectly limits interleukin-10 (IL-10) secretion, which under specific tumor conditions can paradoxically activate CTLs through repression of the Bcl3 cofactor and Bcl3/p50 complex [28]. Moreover, RELA (p65) protects TAMs from T lymphocyte-mediated cytotoxicity by upregulating anti-apoptotic genes including BCL2, MCL1, and BIRC5 (survivin), conferring resistance to host immune attack [28].

Myeloid-specific RELA (p65) deletion results in substantial alterations in the tumor secretome: production of pro-inflammatory cytokines (IFNγ, TNFα) and chemokines (CCL2/MCP-1, CCL3/MIP-1α) increases, promoting mature dendritic cell recruitment and CD8+ lymphocyte proliferation, thereby restoring anti-tumor immunity [27]. Consequently, elevated p65 activation in TAMs positions this subunit as a promising therapeutic target in cancer immunotherapy [27,28].

The current study revealed that patients with higher-grade meningiomas (WHO grades 2 and 3) demonstrated significantly higher *NFKB1* (*p50*) expression in PBMCs compared to patients with lower-grade tumors (WHO grade 1), which may suggest impaired regulatory or signaling mechanisms essential for appropriate immune cell function in response to progressive malignancy [23,29]. While p50/p65 heterodimers typically activate transcription of pro-inflammatory and pro-survival genes, p50/p50 homodimers often function as transcriptional repressors due to the lack of a transactivation domain [24,26,30]. Studies by Li et al. [29] confirm that higher-grade meningiomas induce both local and systemic immunosuppression. Their findings demonstrated that patients with grade 3 meningiomas exhibit elevated PD-L1 expression on peripheral blood monocytes and increased frequencies of myeloid-derived suppressor cells (MDSCs) [29]. Increased *NFKB1* (*p50*) expression in PBMCs may represent a mechanism underlying this observed expansion of suppressor cell populations. The NF-κB pathway is essential for myeloid cell differentiation and function [27,31]. Increased *NFKB1* (*p50*) expression may indicate impaired maturation of effector immune cells, favoring suppressive populations that facilitate tumor immune evasion [23,29]. Higher *NFKB1* (*p50*) expression in PBMCs could potentially serve as a peripheral biomarker to assess tumor grade preoperatively, analogous to the proposed utility of monocyte PD-L1 levels [29]. However, given the limited sample size and exploratory nature of our work, this potential biomarker role should be viewed as preliminary and requires validation in larger, independent cohorts.

In our study, PBMCs from male meningioma patients demonstrated significantly higher *REL* (*c-Rel*) expression compared to female patients. Expression of both catalytic *IKK* complex subunits, *CHUK* (*IKKα*) and *IKBKB* (*IKKβ*), was also significantly elevated in PBMCs from male versus female meningioma patients.

Although meningiomas occur significantly more frequently in women (female-to-male ratio of 2:1 to 3:1), men more often develop aggressive tumor forms (WHO grades 2 and 3) [17,32,33,34]. Male patients exhibit higher probability of harboring meningiomas with malignant methylation profiles and elevated copy number variation (CNV) risk [32]. Elevated NF-κB pathway gene expression in PBMCs may represent a systemic reflection of this increased tumor aggressiveness. Higher *CHUK* (*IKKα*) and *IKBKB* (*IKKβ*) expression in men indicates greater potential for IKK complex activation, essential for canonical NF-κB pathway engagement [31]. Like RELA (p65), c-REL possesses a transactivation domain (TAD) and is considered among the most potent transcriptional activators in the NF-κB family [31]. Elevated *c-REL* expression in PBMCs from male patients may suggest enhanced pro-inflammatory immune activation or differential systemic regulation compared to women. Berghaus et al. [32] noted that sexual dimorphism in meningiomas is frequently overlooked despite sex influencing both pathogenesis and treatment response. Differences in *c-REL*, *CHUK* (*IKKα*), and *IKBKB* (*IKKβ*) expression in PBMCs may therefore serve as sex-specific biomarkers, enabling improved preoperative risk stratification and indicating the need for distinct immunotherapeutic strategies in men and women, reflecting baseline differences in NF-κB pathway activity in immune cells. Again, these sex-related associations should be interpreted as hypothesis-generating and warrant confirmation in prospective studies with adequate power to explore sex-specific effects.

The present cross-sectional design precludes any firm conclusions regarding the directionality of the association between *NF-κB/IKK* transcript changes in PBMCs and meningioma. Similar NF-κB pathway alterations have been described in PBMCs of patients with other chronic inflammatory and neurodegenerative conditions, suggesting that peripheral blood cells can mirror disease-related immune dysregulation [35,36]. On the other hand, tumors are known to shape systemic immunity through cytokines, chemokines, exosomes and other mediators, and NF-κB signaling is a central node in this bidirectional crosstalk within the tumor microenvironment [37,38]. Our data are therefore compatible with both tumor-driven systemic immunomodulation and host-intrinsic immune traits that may influence tumor behavior. Longitudinal and mechanistic studies integrating tumor tissue, tumor-infiltrating immune cells and PBMCs, along with functional NF-κB assays, will be necessary to clarify these relationships.

### Study Limitations

An important limitation of our study is the age discrepancy between the meningioma group (median 64 years) and the control group (median 50 years). NF-κB signaling has been repeatedly linked to aging and “inflammaging”, with elevated NF-κB-dependent transcription described in biologically aged dermal fibroblasts, in aged CD4+ T cells, and across multiple tissues [39,40]. However, within the meningioma cohort, gene expression levels did not differ by age group (≤64 years versus >64 years), and no significant correlations between age and any *NF-κB* or *IKK* transcripts were identified, suggesting that the observed differences are unlikely to be attributable to age alone. The recruitment of age-matched controls was limited by the nature of the disease, as meningioma primarily affects older adults. While basal NF-κB activity in PBMCs typically increases with age (associated with “inflammaging”) [39], we observed a significant decrease in *NFKB1* expression in our older meningioma patient cohort. This suggests that the tumor-associated immunomodulation may override normal age-related trends; nevertheless, age remains a potential confounding variable that cannot be fully excluded. Future studies should therefore employ strictly age- and sex-matched control cohorts to better separate tumor-related immune alterations from age-associated changes in NF-κB signaling.

The second study limitation is the relatively small cohort size (*n* = 31 patients; *n* = 18 controls), which substantially limits statistical power, particularly for subgroup analyses such as those comparing WHO grade G2/G3 patients (*n* = 5) with G1 patients (*n* = 26). The relatively small sample size reflects the rarity of meningiomas and the inherent challenges of patient recruitment within a single-center setting. Accordingly, we explicitly consider this work an exploratory pilot study, and the findings should be viewed as hypothesis-generating rather than definitive. However, we would like to emphasize that our research is preliminary in nature and undoubtedly requires validation in an independent cohort.

Finally, this first exploratory analysis of NF-κB pathway components in PBMCs of meningioma patients demonstrates heterogeneous statistical power across the analyzed genes. Adequate power was achieved for *NFKB1*, *RELA*, *CHUK*, and *IKBKB*, supporting the robustness of the observed differences for these targets. In contrast, substantially larger sample sizes would be required to reliably assess *NFKB2*, *RELB*, *c-REL*, and *IKBKG*, and therefore findings related to these genes should be interpreted with caution. Taken together, these limitations underscore the preliminary nature of our observations and highlight the need for validation in larger, multicenter and adequately powered cohorts encompassing more diverse patient populations.

## 5. Conclusions

In summary, our pilot study may suggest NF-κB pathway dysregulation in PBMCs from meningioma patients, potentially characterized by decreased *NFKB1 (p50)* expression and increased *RELA (p65)*, *CHUK (IKKα)*, and *IKBKB (IKKβ)* expression compared to healthy controls. The reduction in *NFKB1* expression appeared to be more pronounced in higher WHO grades (G2 and G3), which may indicate that *NFKB1* expression could warrant further investigation as a candidate peripheral biomarker for preoperative tumor aggressiveness assessment. However, given the small cohort and cross-sectional design, this potential biomarker role should be regarded as preliminary. These alterations might reflect a shift in dimerization equilibrium toward p65/p65 homodimers and activation of the canonical pathway, which has been hypothesized to contribute to systemic immunosuppression in analogous settings, tumor cell survival, and impaired effector immune cell maturation. Because our data are restricted to mRNA expression, we cannot directly demonstrate such changes at the protein or functional level, and the mechanistic scenarios discussed here should therefore be tested in future studies integrating protein-level and functional assays of NF-κB activity. We also observed sexual dimorphism, with elevated *c-REL* and *IKK* complex subunit expression in male patients, which may suggest that sex could represent a relevant factor, requiring prospective confirmation. Collectively, our findings may indicate that peripheral dysregulation of the p65 subunit and the broader NF-κB pathway could also be present in peripheral mononuclear cells and may contribute to, or at least reflect, meningioma progression; this hypothesis warrants further mechanistic and longitudinal investigation.

## Figures and Tables

**Table 1:** Demographic, clinical, and routine laboratory results characteristics of meningioma tumor patients. Results are presented as the number of cases and a percentage or as a median with 25th and 75th percentiles.

Variable	Meningioma Tumor Group No. 31
Gender, F/M	21 (68%)/10 (32%)
Age, yr	64 (52–71)
Histopathological type	
Fibrous	10 (33%)
Meningothelial	8 (26%)
Angiomatous	5 (16%)
Transitional	3 (10%)
Rhabdoid	2 (6%)
Anaplastic	1 (3%)
Atypic	1 (3%)
Metaplastic	1 (3%)
WHO grade	
G1	26 (84%)
G2	2 (6%)
G3	3 (10%)
WBC [10^3^/μL]	7.61 (6.78–9.19)
RBC [10^6^/μL]	4.25 (4.00–4.72)
HGB [g/dL]	13.2 (12.3–14.3)
HCT [%]	38.3 (36.1–41.9)
MCV [fL]	90.0 (86.9–91.4)
PLT [10^3^/μL]	217 (183–268)
MPV [fL]	10.3 (9.7–11.2)
P-LCR [%]	28.5 (22.0–35.2)
PT [s]	13.2 (12.7–13.7)
INR	1.00 (0.95–1.04)
APTT [s]	26.9 (25.4–28.3)
APTT ratio	0.92 (0.87–0.97)
Fibrinogen [mg/dL]	335 (253–370)
Glucose [mg/dL]	104 (86–128)
Urea [mg/dL]	31.83 (23.00–38.52)
Creatinine [mg/dL]	0.71 (0.62–0.82)
eGFR [mL/min]	100 (86–111)
Na [mmol/L]	140 (137–141)
K [mmol/L]	4.1 (3.9–4.4)
SI conversion factors: WBC to ×10^9^/L, multiply by 1.0; RBC to ×10^12^/L, multiply by 1.0; HGB to g/L, multiply by 10.0; HCT to proportion of 1.0, multiply by 0.01; PLT to ×10^9^/L, multiply by 1.0; fibrinogen to g/L, multiply by 0.01; glucose to mmol/L, multiply by 0.0555; creatinine to mmol/L, multiply by 0.0884; urea to mmol/L, multiply by 0.1665.	

Legend for Table 1: F—female; M—male; yr—years; WBC—white blood cell; RBC—red blood cell; HGB—hemoglobin concentration; HCT—hematocrit; MCV—mean corpuscular volume; PLT—platelets; MPV—mean platelet volume, P-LCR—platelet large cell ratio; PT—prothrombin time; INR—International Normalized Ratio; APTT—activated partial thromboplastin time; eGFR—estimated glomerular filtration rate; Na—sodium; K—potassium.

## Data Availability

The original contributions presented in this study are included in the article. Further inquiries can be directed to the corresponding authors.

## Supplementary Materials

The following supporting information can be downloaded at: https://www.mdpi.com/article/10.3390/life16060880/s1. Figure S1. Comparison of *NF-κB* isoforms and *IKK* subunits expression in PBMCs of meningioma patients according to histopathological classification. (A) Δ*NFKB1*; (B) Δ*NFKB2*; (C) Δ*RELA*; (D) Δ*RELB*; (E) Δ*c-REL*; (F) Δ*CHUK*; (G) Δ*IKBKB*; (H) Δ*IKBKG*. Cycle threshold (Ct) values obtained from technical triplicates were averaged. Relative gene expression levels were calculated using the ΔCt method, where ΔCt represents the difference between the mean Ct of the target gene and the mean Ct of the reference gene (*GAPDH*). Box-and-whisker plots display interquartile ranges with median values, and whiskers indicate the full data range (minimum to maximum). IKK—I kappa B kinase; PBMCs—peripheral blood mononuclear cells; NFKB1—nuclear factor kappa B subunit 1; NFKB2—nuclear factor kappa B subunit 2; RELA—RELA Proto-Oncogene; RELB—RELB Proto-Oncogene; c-REL—REL Proto-Oncogene; CHUK—conserved helix-loop-helix ubiquitous kinase; IKBKB—inhibitor of nuclear factor kappa B kinase subunit beta; IKBKG—inhibitor of nuclear factor kappa B kinase subunit gamma; Figure S2. Comparison of *NF-κB* isoforms and *IKK* subunits expression in PBMCs of meningioma patients according to median age. (A) Δ*NFKB1*; (B) Δ*NFKB2*; (C) Δ*RELA*; (D) Δ*RELB*; (E) Δ*c-REL*; (F) Δ*CHUK*; (G) Δ*IKBKB*; (H) Δ*IKBKG*. Cycle threshold (Ct) values obtained from technical triplicates were averaged. Relative gene expression levels were calculated using the ΔCt method, where ΔCt represents the difference between the mean Ct of the target gene and the mean Ct of the reference gene (*GAPDH*). Box-and-whisker plots display interquartile ranges with median values, and whiskers indicate the full data range (minimum to maximum). A *p*-value of <0.05 (two-tailed) was considered statistically significant. IKK—I kappa B kinase; PBMCs—peripheral blood mononuclear cells; NFKB1—nuclear factor kappa B subunit 1; NFKB2—nuclear factor kappa B subunit 2; RELA—RELA Proto-Oncogene; RELB—RELB Proto-Oncogene; c-REL—REL Proto-Oncogene; CHUK—conserved helix-loop-helix ubiquitous kinase; IKBKB—inhibitor of nuclear factor kappa B kinase subunit beta; IKBKG—inhibitor of nuclear factor kappa B kinase subunit gamma; Table S1. Comparison of the routine laboratory examination results between meningioma female patients and meningioma male patients. Results are presented as median with 25th and 75th percentiles. Table S2. Comparison of the routine laboratory examination results depending on the tumor grade in meningioma patients. Results are presented as median with 25th and 75th percentiles. Table S3. Comparison of the routine laboratory examination results depending on the median age of meningioma patients. Results are presented as median with 25th and 75th percentiles. Table S4. Comparison of the routine laboratory examination results depending on the histopathological type of meningioma. Results are presented as median with 25th and 75th percentiles. Table S5. Correlation coefficient results for *NFKB* isoforms and *IKK* complex subunits in PBMCs from meningioma patients; Table S6. Sample size and post-hoc power analysis for NFKB1, NFKB2, RELA, RELB, c-REL, CHUK, IKBKB, and IKBKG in PBMCs when comparing meningioma patients group and control subjects.
