# Supplementary material for: Expression of NF-κB Isoforms and IKK Complex Subunits Differs in Peripheral Blood Mononuclear Cells (PBMCs) of Patients with Meningiomas: A Pilot Study
Source: Life (Basel). 2026 May 24;16(6):880. doi: 10.3390/life16060880 (PMC13301613; doi:10.3390/life16060880)
Supplement: Supplementary file 1 [file life-16-00880-s001.zip › Tables S1-S6.pdf]

Table S1. Comparison of the routine laboratory examination results between meningioma female patients and meningioma male patients. Results are presented as median with 25th and 75th percentiles.

Variable	Females No. 21	Males No. 10	p-value
WBC [$10^3/\mu L$]	7.46 (6.54-8.28)	8.67 (7.06-10.12)	0.1354
RBC [$10^6/\mu L$]	4.25 (4.03-4.34)	4.74 (3.99-4.93)	0.1864
HGB [g/dL]	13.1 (12.7-13.6)	15.1 (12.3-15.6)	0.1354
HCT [%]	38.2 (36.2-39.7)	43.3 (35.2-44.8)	0.1594
MCV [fL]	89.5 (86.8-91.3)	90.9 (88.0-91.4)	0.5189
PLT [$10^3/\mu L$]	227 (199-267)	200 (181-270)	0.4409
MPV [fL]	10.4 (9.6-11.1)	10.2 (9.8-11.5)	0.7872
P-LCR [%]	28.5 (21.9-34.7)	28.3 (24.1-36.5)	0.6032
PT [s]	13.2 (12.8-13.6)	13.3 (12.6-14.2)	0.4921
INR	1.00 (0.95-1.03)	1.02 (0.94-1.07)	0.5745
APTT [s]	26.9 (25.1-28.1)	26.3 (25.7-28.3)	0.8193
APTT ratio	0.92 (0.86-0.96)	0.90 (0.88-0.97)	0.7872
Fibrinogen [mg/dL]	335 (276-363)	353 (253-405)	0.6625
Na [mmol/L]	139 (137-141)	141 (138-143)	0.1594
K [mmol/L]	4.10 (3.99-4.25)	4.30 (3.90-4.70)	0.2499
Glucose [mg/dL]	100 (89-128)	110 (85-123)	0.9173
Urea [mg/dL]	33.00 (23.00-38.52)	30.90 (23.54-34.24)	0.9173
Creatinine [mg/dL]	0.66 (0.61-0.74)	0.82 (0.77-0.84)	**0.0077***
eGFR [mL/min]	98 (83-117)	107 (97-110)	0.3927

SI conversion factors: WBC to x10^9/L multiply by 1.0; RBC to x10^{12}/L multiply by 1.0; HGB to g/L multiply by 10.0; HCT to proportion of 1.0 multiply by 0.01; PLT to x10^9/L multiply by 1.0; glucose to mmol/L multiply by 0.0555; creatinine to mmol/L multiply by 0.0884; urea to mmol/L multiply by 0.1665

Legend for Table S1: WBC – white blood cells; RBC – red blood cell; HGB – hemoglobin concentration; HCT – hematocrit; MCV – mean corpuscular volume; PLT – platelets; MPV – mean platelet volume; P-LCR – platelet large cell ratio; PT – prothrombin time; INR – International Normalized Ratio; APTT – activated partial thromboplastin time; Na – sodium; K – potassium; eGFR – estimated glomerular filtration rate; *And bold of a 2-tailed p-value – the level of significance < 0.05.

Table S2. Comparison of the routine laboratory examination results depending on the tumor grade in meningioma patients. Results are presented as median with 25th and 75th percentiles.

Variable	WHO grade G1 No. 26	WHO grade G2+G3 No. 5	p-value
WBC [$10^3/\mu L$]	7.75 (6.95-9.19)	6.82 (6.54-8.21)	0.3870
RBC [$10^6/\mu L$]	4.27 (3.99-4.72)	4.25 (4.23-4.31)	0.7747
HGB [g/dL]	13.2 (12.3-14.3)	13.1 (12.9-13.2)	0.9378
HCT [%]	38.9 (35.2-41.9)	38.2 (36.2-38.3)	0.9378
MCV [fL]	90.2 (88.0-92.0)	88.90 (86.40-90.0)	0.2142
PLT [$10^3/\mu L$]	221 (183-268)	216 (192-227)	0.6203
MPV [fL]	10.3 (9.7-11.2)	10.5 (9.4-10.6)	0.8555
P-LCR [%]	28.3 (22.3-35.2)	29.0 (20.8-29.3)	0.9378
PT [s]	13.2 (12.8-13.7)	12.8 (12.6-13.4)	0.2805
INR	1.0 (0.95-1.05)	0.99 (0.92-1.02)	0.3311
APTT [s]	26.9 (25.7-28.3)	26.3 (25.1-27.5)	0.4802
APTT ratio	0.92 (0.88-0.97)	0.90 (0.86-0.94)	0.4479
Fibrinogen [mg/dL]	336 (253-367)	309 (303-370)	0.9793
Na [mmol/L]	139 (137-141)	141 (140-142)	0.3870
K [mmol/L]	4.2 (4.0-4.40)	3.7 (3.2-4.1)	0.1288
Glucose [mg/dL]	99 (86-123)	151 (128-156)	**0.0413***
Urea [mg/dL]	32.55 (23.54-38.52)	19.26 (19.26-23.54)	0.1288
Creatinine [mg/dL]	0.73 (0.66-0.83)	0.52 (0.39-0.66)	**0.0188***
eGFR [mL/min]	99 (84-107)	125 (109-170)	**0.0223***

SI conversion factors: WBC to x109/L multiply by 1.0; RBC to x1012/L multiply by 1.0; HGB to g/L multiply by 10.0; HCT to proportion of 1.0 multiply by 0.01; PLT to x109/L multiply by 1.0; glucose to mmol/L multiply by 0.0555; creatinine to mmol/L multiply by 0.0884; urea to mmol/L multiply by 0.1665

Legend for Table S2: WBC – white blood cells; RBC – red blood cell; HGB – hemoglobin concentration; HCT – hematocrit; MCV – mean corpuscular volume; PLT – platelets; MPV – mean platelet volume; P-LCR – platelet large cell ratio; PT – prothrombin time; INR – International

Normalized Ratio; APTT – activated partial thromboplastin time; Na – sodium; K – potassium; eGFR – estimated glomerular filtration rate; *And bold of a 2-tailed p-value – the level of significance < 0.05.

Table S3. Comparison of the routine laboratory examination results depending on the median age of meningioma patients. Results are presented as median with 25th and 75th percentiles.

Variable	≤ 64 years No. 16	> 64 years No. 15	p-value
WBC [10^3/μL]	8.19 (7.31-9.27)	7.06 (6.54-8.28)	0.3378
RBC [10^6/μL]	4.31 (4.04-4.73)	4.24 (3.99-4.56)	0.6823
HGB [g/dL]	13.2 (12.5-14.9)	13.1 (11.8-14.1)	0.6823
HCT [%]	38.9 (36.5-43.0)	38.2 (36.1-41.2)	0.5717
MCV [fL]	90.7 (87.8-93.0)	89.5 (86.8-91.0)	0.2475
PLT [10^3/μL]	246 (185-285)	216 (183-249)	0.2164
MPV [fL]	10.6 (9.9-11.5)	9.9 (9.4-10.6)	0.1195
P-LCR [%]	29.1 (24.3-36.3)	23.4 (20.0-29.3)	0.1195
PT [s]	13.3 (12.7-13.9)	12.9 (12.7-13.6)	0.4463
INR	1.0 (0.95-1.06)	0.99 (0.95-1.03)	0.5717
APTT [s]	26.9 (25.7-28.6)	26.9 (25.1-28.1)	0.8304
APTT ratio	0.92 (0.88-0.98)	0.92 (0.86-0.96)	0.8304
Fibrinogen [mg/dL]	335 (263-397)	336 (253-370)	0.8304
Na [mmol/L]	140 (138-142)	140 (137-141)	0.8001
K [mmol/L]	4.20 (4.10-4.40)	4.10 (3.70-4.40)	0.2995
Glucose [mg/dL]	90 (84-104)	123 (98-150)	**0.0171***
Urea [mg/dL]	27.82 (21.13-35.31)	33.0 (23.54-42.80)	0.4008
Creatinine [mg/dL]	0.74 (0.66-0.82)	0.71 (0.60-0.83)	0.4945
eGFR [mL/min]	104 (93-111)	94 (83-125)	0.4701

SI conversion factors: WBC to x109/L multiply by 1.0; RBC to x1012/L multiply by 1.0; HGB to g/L multiply by 10.0; HCT to proportion of 1.0 multiply by 0.01; PLT to x109/L multiply by 1.0; glucose to mmol/L multiply by 0.0555; creatinine to mmol/L multiply by 0.0884; urea to mmol/L multiply by 0.1665

Legend for Table S3: WBC – white blood cells; RBC – red blood cell; HGB – hemoglobin concentration; HCT – hematocrit; MCV – mean corpuscular volume; PLT – platelets; MPV – mean platelet volume; P-LCR – platelet large cell ratio; PT – prothrombin time; INR – International

Normalized Ratio; APTT – activated partial thromboplastin time; Na – sodium; K – potassium; eGFR – estimated glomerular filtration rate; *And bold of a 2-tailed p-value – the level of significance < 0.05.

Table S4. Comparison of the routine laboratory examination results depending on the histopathological type of meningioma. Results are presented as median with 25th and 75th percentiles.

Variable	Fibrous No. 10	Meningothelial No. 8	Angiomatous No. 5	p-value
WBC [10^3/μL]	8.14 (7.46-10.62)	7.17 (6.58-8.03)	6.78 (5.65-7.34)	0.1383
RBC [10^6/μL]	4.25 (4.0-4.48)	4.02 (3.50-4.54)	4.34 (4.28-4.72)	0.6335
HGB [g/dL]	12.9 (11.8-14.0)	12.3 (10.8-14.0)	13.9 (13.5-14.3)	0.6816
HCT [%]	38.2 (36.2-40.0)	35.7 (31.6-40.8)	41.8 (39.7-41.9)	0.5598
MCV [fL]	87.9 (86.2-91.3)	90.3 (89.6-91.4)	88.6 (88.1-92.8)	0.4224
PLT [10^3/μL]	237.0 (174.0-263.0)	211.50 (182.0-246.50)	267.0 (201.0-304.0)	0.5518
MPV [fL]	9.7 (9.4-11.7)	10.6 (10.2-11.3)	10.3 (9.8-10.4)	0.3126
P-LCR [%]	22.7 (20.0-39.1)	29.1 (26.1-34.7)	27.0 (22.3-28.5)	0.3209
PT [s]	13.4 (12.8-14.1)	13.0 (12.5-13.3)	12.9 (12.8-12.9)	0.3707
INR	1.02 (0.95-1.09)	0.99 (0.94-1.02)	0.96 (0.95-0.99)	0.2184
APTT [s]	27.2 (26.9-28.3)	26.8 (25.8-28.0)	27.0 (25.1-27.6)	0.7904
APTT ratio	0.93 (0.92-0.97)	0.92 (0.89-0.96)	0.93 (0.86-0.95)	0.8685
Fibrinogen [mg/dL]	340.0 (308.0-363.0)	353.0 (294.50-432.0)	333.0 (240.0-333.0)	0.3065
Na [mmol/L]	140.0 (139.0-141.0)	141.0 (137.50-142.0)	139.0 (138.0-139.0)	0.2860
K [mmol/L]	4.1 (3.9-4.20)	4.5 (4.0-4.7)	4.3 (4.2-4.3)	0.1462
Glucose [mg/dL]	114 (98-128)	102 (86-119)	89 (86-96)	0.3855
Urea [mg/dL]	37.45 (23.54-48.0)	30.90 (28.89-33.17)	33.0 (27.82-34.24)	0.3596
Creatinine [mg/dL]	0.69 (0.62-0.80)	0.81 (0.74-0.89)	0.68 (0.66-0.71)	0.1896
eGFR [mL/min]	94 (83-111)	96 (84-109)	98 (88-101)	0.9665

SI conversion factors: WBC to x109/L multiply by 1.0; RBC to x1012/L multiply by 1.0; HGB to g/L multiply by 10.0; HCT to proportion of 1.0 multiply by 0.01; PLT to x109/L multiply by 1.0; glucose to mmol/L multiply by 0.0555; creatinine to mmol/L multiply by 0.0884; urea to mmol/L multiply by 0.1665

Legend for Table S4: WBC – white blood cells; RBC – red blood cell; HGB – hemoglobin concentration; HCT – hematocrit; MCV – mean corpuscular volume; PLT – platelets; MPV – mean platelet volume; P-LCR – platelet large cell ratio; PT – prothrombin time; INR – International Normalized Ratio; APTT – activated partial thromboplastin time; Na – sodium; K – potassium; eGFR – estimated glomerular filtration rate; *And bold of a 2-tailed p-value – the level of significance < 0.05.

Table S5. Correlation coefficient results for *NFKB* isoforms and *IKK* complex subunits in PBMCs from meningioma patients.

	Age	NFKB1	NFKB2	RELA	RELB	c-REL	CHUK	IKBKB	IKBKG
Age	-	R=0.14; p=0.4396	R= -0.07; p=0.7343	R=0.05; p=0.7774	R= -0.11; p=0.5625	R=0.02; p=0.9330	R=0.16; p=0.3935	R=0.05; p=0.7749	R=0.04; p=0.8199
NFKB1	R=0.14; p=0.4396	-	**R=0.49; p=0.0070**	R= -0.01; p=0.9725	R= -0.06; p=0.7516	**R=0.62; p=0.0002**	**R=0.53; p=0.0027**	**R=0.45; p=0.0109**	R=0.15; p=0.4105
NFKB2	R= -0.07; p=0.7343	**R=0.49; p=0.0070**	-	**R=0.55; p=0.0019**	R=0.22; p=0.2606	**R= -0.49; p=0.0065**	R=0.29; p=0.1323	**R=0.44; p=0.0159**	R=0.20; p=0.2862
RELA	R=0.05; p=0.7774	R= -0.01; p=0.9725	**R=0.55; p=0.0019**	-	R=0.11; p=0.5482	**R=0.44; p=0.0132**	R=0.28; p=0.1293	**R=0.37; p=0.0388**	R=0.35; p=0.0560
RELB	R= -0.11; p=0.5625	R= -0.06; p=0.7516	R=0.22; p=0.2606	R=0.11; p=0.5482	-	R=0.10; p=0.6142	R=-0.08; p=0.6910	R=0.02; p=0.9043	R= -0.28; p=0.1326
c-REL	R=0.02; p=0.9330	**R=0.62; p=0.0002**	**R= -0.49; p=0.0065**	**R=0.44; p=0.0132**	R=0.10; p=0.6142	-	**R=0.67; p=0.0001**	**R=0.68; p<0.0001**	**R=0.39; p=0.0287**
CHUK	R=0.16; p=0.3935	**R=0.53; p=0.0027**	R=0.29; p=0.1323	R=0.28; p=0.1293	R= -0.08; p=0.6910	**R=0.67; p=0.0001**	-	**R=0.84; p<0.0001**	**R=0.57; p=0.0011**
IKBKB	R=0.05; p=0.7749	**R=0.45; p=0.0109**	**R=0.44; p=0.0159**	**R=0.37; p=0.0388**	R=0.02; p=0.9043	**R=0.68; p<0.0001**	**R=0.84; p<0.0001**	-	**R=0.75; p<0.0001**
IKBKG	R=0.04; p=0.8199	R=0.15; p=0.4105	R=0.20; p=0.2862	R=0.35; p=0.0560	R= -0.28; p=0.1326	**R=0.39; p=0.0287**	**R=0.57; p=0.0011**	**R=0.75; p<0.0001**	-

Legend for Table S5: PBMCs – peripheral blood mononuclear cells; NFKB1 – nuclear factor kappa B subunit 1; NFKB2 – nuclear factor kappa B subunit 2; RELA – RELA Proto-Oncogene; RELB – RELB Proto-Oncogene; c-REL – REL Proto-Oncogene; CHUK – conserved helix-loop-helix ubiquitous kinase; IKBKB – inhibitor of nuclear factor kappa B kinase subunit beta; IKBKG – inhibitor of nuclear factor kappa B kinase regulatory subunit gamma; R – correlation coefficient; p – p-value.

Table S6. Sample size and post-hoc power analysis for *NFKB1, NFKB2, RELA, RELB, c-REL, CHUK, IKBKB,* and *IKBKG* in PBMCs when comparing meningioma patients group and control subjects.

Parameter	Sample size (R)	Sample size (CT)	Post-hoc power analysis [%] (R)
NFKB1	4188	12	96 (3.8)
NFKB2	1501	487	7.5 (4.5)
RELA	351	4	100 (8.4)
RELB	1704	12016	3.2 (5.8)
c-REL	23876	75	27.5 (3)
CHUK	56512	11	99 (2.9)
IKBKB	34676	8	100 (3)
IKBKG	8449	1539	5.2 (3.5)

Legend for Table S6: *NFKB1* – nuclear factor kappa B subunit 1; *NFKB2* – nuclear factor kappa B subunit 2; *RELA* – RELA Proto-Oncogene; *RELB* – RELB Proto-Oncogene; *c-REL* – REL Proto-Oncogene; CHUK – conserved helix-loop-helix ubiquitous kinase; IKBKB – inhibitor of nuclear factor kappa B kinase subunit beta; IKBKG – inhibitor of nuclear factor kappa B kinase regulatory subunit gamma
