# Supplementary figures and images for: Expression of NF-κB Isoforms and IKK Complex Subunits Differs in Peripheral Blood Mononuclear Cells (PBMCs) of Patients with Meningiomas: A Pilot Study
Source: Life (Basel). 2026 May 24;16(6):880. doi: 10.3390/life16060880 (PMC13301613; doi:10.3390/life16060880)

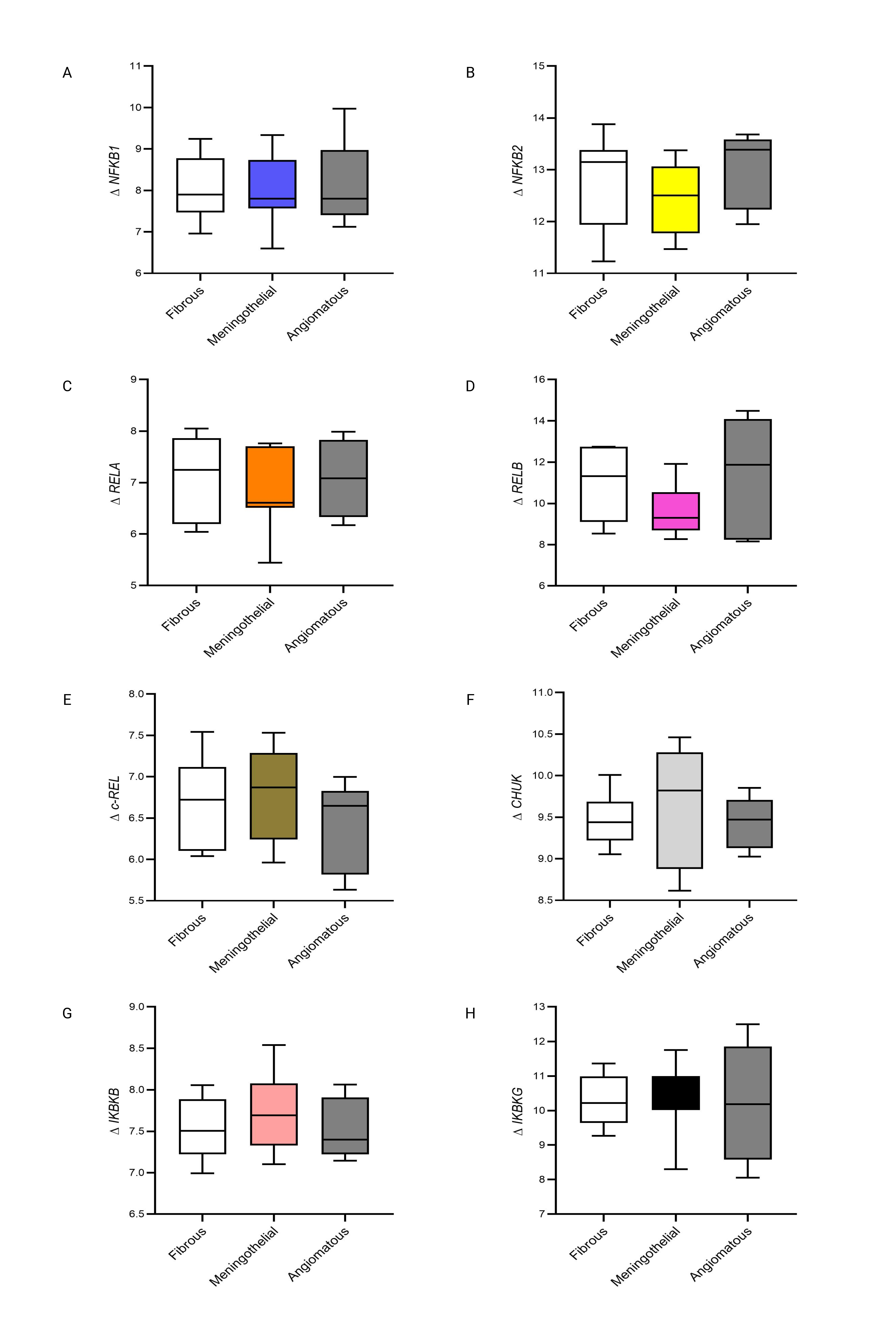

Supplement: Supplementary file 1 [file life-16-00880-s001.zip › Figure S1.tiff]

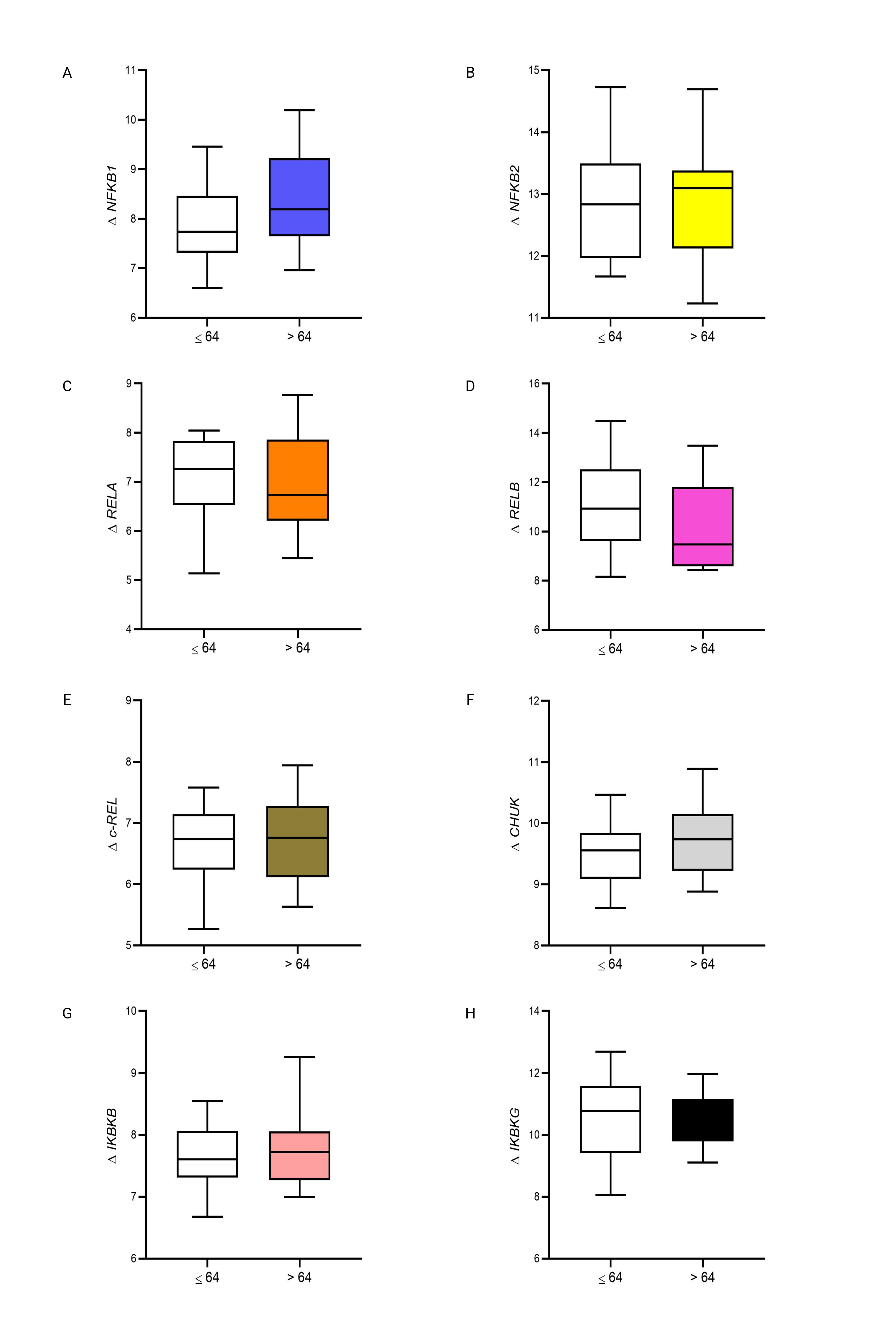

Supplement: Supplementary file 1 [file life-16-00880-s001.zip › Figure S2.tiff]
